# Development of machine learning models integrating PET/CT radiomic and immunohistochemical pathomic features for treatment strategy choice of cervical cancer with negative pelvic lymph node by mediating COX-2 expression

**DOI:** 10.3389/fphys.2022.994304

**Published:** 2022-10-14

**Authors:** Zhe Zhang, Xiaoran Li, Hongzan Sun

**Affiliations:** Department of Radiology, Shengjing Hospital of China Medical University, Shenyang, China

**Keywords:** PET/CT, lymphatic metastasis, radiomics, cyclooxygenase 2, machine learning

## Abstract

**Objectives:** We aimed to establish machine learning models based on texture analysis predicting pelvic lymph node metastasis (PLNM) and expression of cyclooxygenase-2 (COX-2) in cervical cancer with PET/CT negative pelvic lymph node (PLN).

**Methods:** Eight hundred and thirty-seven texture features were extracted from PET/CT images of 148 early-stage cervical cancer patients with negative PLN. The machine learning models were established by logistic regression from selected features and evaluated by the area under the curve (AUC). The correlation of selected PET/CT texture features predicting PLNM or COX-2 expression and the corresponding immunohistochemical (IHC) texture features was analyzed by the Spearman test.

**Results:** Fourteen texture features were reserved to calculate the Rad-score for PLNM and COX-2. The PLNM model predicting PLNM showed good prediction accuracy in the training and testing dataset (AUC = 0.817, *p* < 0.001; AUC = 0.786, *p* < 0.001, respectively). The COX-2 model also behaved well for predicting COX-2 expression levels in the training and testing dataset (AUC = 0.814, *p* < 0.001; AUC = 0.748, *p* = 0.001). The wavelet-LHH-GLCM ClusterShade of the PET image selected to predict PLNM was slightly correlated with the corresponding feature of the IHC image (r = −0.165, *p* < 0.05). There was a weak correlation of wavelet-LLL-GLRLM LongRunEmphasis of the PET image selected to predict COX-2 correlated with the corresponding feature of the IHC image (r = 0.238, *p* < 0.05). The correlation between PET image selected to predict COX-2 and the corresponding feature of the IHC image based on wavelet-LLL-GLRLM LongRunEmphasis is considered weak positive (r = 0.238, *p*=<0.05).

**Conclusion:** This study underlined the significant application of the machine learning models based on PET/CT texture analysis for predicting PLNM and COX-2 expression, which could be a novel tool to assist the clinical management of cervical cancer with negative PLN on PET/CT images.

## Introduction

Cervical cancer is the fourth most prevalent cancer and the fourth leading cause of female cancer, with more than 6,00,000 incidences and 3,00,000 death cases reported in 2020 (Sung et al., 2021). The most commonly used clinical treatments are radical hysterectomy with pelvic lymph node dissection. However, the prognosis of post-operative patients varied significantly due to tumor heterogeneity (Bhatla et al., 2021). Several studies have indicated the most relevant prognostic factor in early cervical cancer is pelvic lymph node metastasis (PLNM) (Rudtanasudjatum et al., 2011; Horn et al., 2014; Zyla et al., 2020; Federico et al., 2022; Wenzel et al., 2022). Kulisara et al. have proved that patients with lymph node metastasis (LNM) had poorer 5-year overall survival than the patients without LNM (*p* < 0.05) (Nanthamongkolkul and Hanprasertpong, 2018). In addition, lymphadenectomy may increase the probability of some complications including lower limb lymphedema, ileus, and chylous ascites (Yost et al., 2014; Kuroda et al., 2017; Nica et al., 2020; Umbreit et al., 2020). The accuracy of predicting LNM in cervical cancer patients is crucial for treatment decision-making.

Previous studies have indicated that PET/CT could be used for the evaluation of LNM as a preoperative imaging test, which is of vital importance for clinical strategies and individualized treatment (Sironi et al., 2006; Lv et al., 2014; Fasmer et al., 2020). Nevertheless, these studies usually used lymph node metabolism and diameter to assess LNM. Few studies have evaluated the metastasis of PLN with slightly higher FDG metabolism and diameter less than 1 cm on PET images which has limitations in detecting micrometastasis. Radiomics is rapidly gaining momentum and this technique is characterized by quantifying tumor heterogeneity through extraction of computational features using advanced computational algorithms. Texture parameters of radiomics features of PET images and IHC pathomic features could potentially be adopted to predict the PLNM for strategy choice of cervical cancer patients.

Substantial evidence suggests that cyclooxygenase-2 (COX-2), a key protein in prostaglandin metabolism, has a critical role in PLNM in cervical cancer (Ryu et al., 2000; Hoellen et al., 2016). Previous studies have indicated elevated COX-2 was strongly related to LNM in stage IB cervical cancer (Kang et al., 2006), the high COX expression has been revealed positive correlation with malignancy in the parametrial tumor tissue or LNM (Ryu et al., 2000). Other studies also found that high-level expression of COX-2 was correlated with a poorer prognosis, recurrence, low sensitivity of nedaplatin, and radiosensitivity (Kim et al., 2003; Chen et al., 2005; Ishikawa et al., 2006; Manchana et al., 2006; Jo et al., 2007; Huang et al., 2013; Stasinopoulos et al., 2013; Kato et al., 2015). In neoplasia, COX-2 stimulates cell proliferation which promotes angiogenesis through pathways involving an increase in VEGF production (Huang et al., 2013; Xu et al., 2014). It has been suggested that COX-2 expression may enhance LNM after the onset of lymphovascular space invasion (Khunamornpong et al., 2009; Hoellen et al., 2016). The heterogenic ^18^F-FDG uptake was strongly related to the histopathological appearance in the tumor region. ^18^F-FDG heterogenic uptake within the tumor was correlated with the heterogeneity of tumor histopathological tissues (Zhao et al., 2005; Henriksson et al., 2007). IHC assay demonstrated that tumor angiogenesis and cancer cell proliferation were significantly related to the enhancement of tumor heterogeneity. Therefore, the high expression of COX-2 played a connecting role between the increase of tumor heterogeneity and PLNM (Liu et al., 2011).

Based on texture parameters of radiomics features of PET images, the global and local-regional heterogeneities of ^18^F-FDG distribution could be potentially assessed. Moreover, some mathematical methods were obtained to describe the relationships between their position in PET images and the gray-level intensity of pixels or voxels (Chicklore et al., 2013; O’Connor, 2017). In this study, we hypothesized that the overexpression of COX-2 promoted the increase of tumor heterogeneity and then caused the change of texture features of radiomics derived from IHC and PET/CT imaging. The texture features of the primary tumor lesion may be correlated with PLNM in patients with early-stage cervical cancer. Therefore, we aimed to establish machine learning models of texture analysis that could predict PLNM and COX-2 expression based on PET/CT imaging to assist the clinical management of PLNM therapy in cervical cancer with PET/CT negative PLN.

## Materials and methods

### Radiomics workflow

**FIGURE 1:**
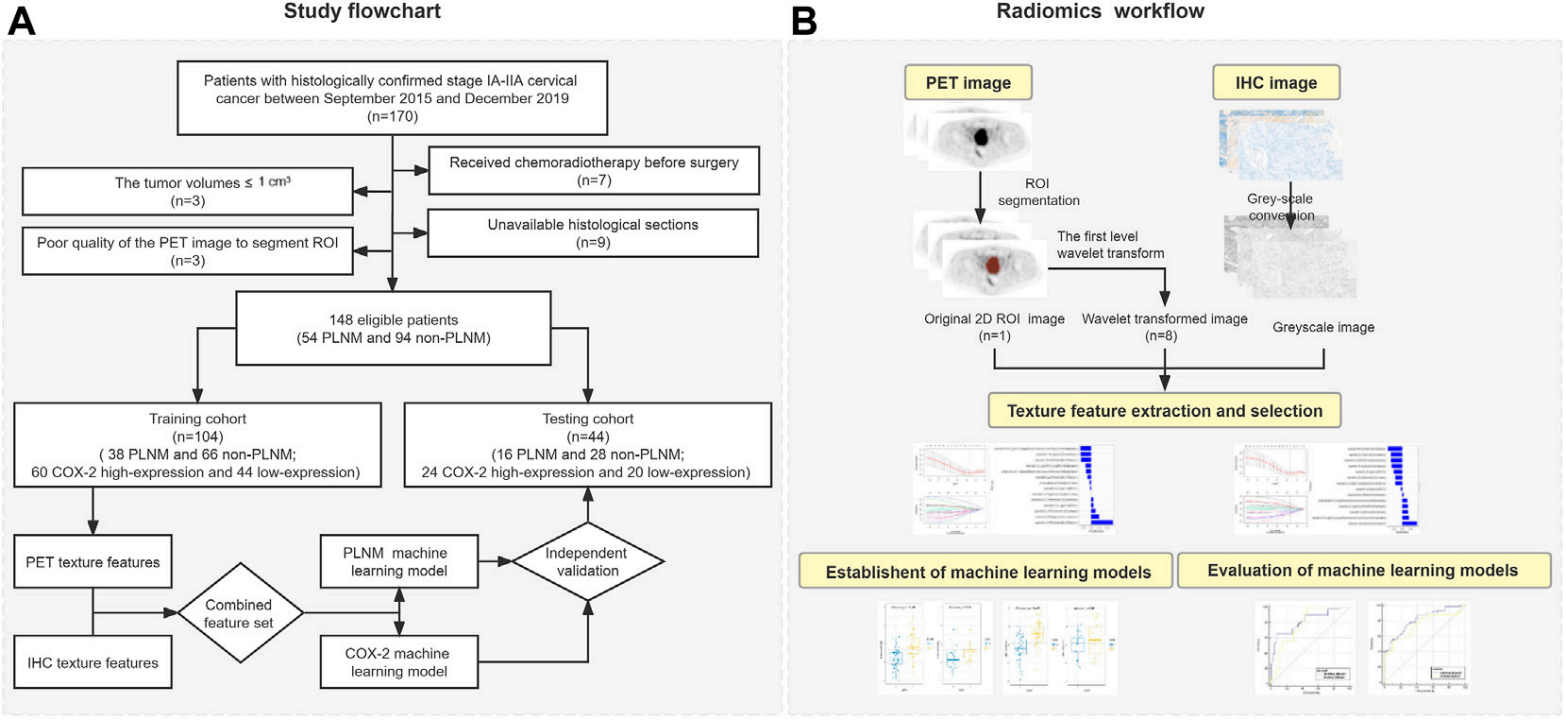
Study flowchart and radiomics workflow. **(A)** Study flowchart. **(B)** Radiomics workflow.

The study flowchart and radiomics workflow are shown in Figure 1 and includes the collection and exclusion of patients, image acquisition, ROI segmentation, feature extraction and selection, establishment and evaluation of machine learning models, and correlation analysis between PET and IHC images with the same texture features.

### Patients

This retrospective study consisted of 170 patients with histologically confirmed stage IA-IIA cervical cancer who underwent radical hysterectomy with pelvic node dissection between September 2015 and December 2019 in Shengjing Hospital of China Medical University. All patients underwent ^18^F-FDG PET/CT scan within 1 week before treatment. The Hospital Institutional Review Board approved this study and informed consents were nor required due to retrospective nature. Inclusion criteria for cases: (Sung et al., 2021) Cervical squamous cell carcinoma confirmed by pathology and lymph node dissection performed in the patient; (Bhatla et al., 2021) Ia-IIa stage identified by 2021 Federation of Gynecology and Obstetrics (FIGO) staging (Bhatla et al., 2021); (Horn et al., 2014) The SUV_max_ of PLNM was less than 2.5 and the diameter was less than 1 cm; (Zyla et al., 2020) Normal serum glucose level before PET/CT scanning; (Rudtanasudjatum et al., 2011) No other tumor or metabolic disease. A total of twenty-two patients were excluded from the sample. Seven of them were excluded because they had received chemoradiotherapy before surgery. Nine histological sections could not be obtained. The tumor volumes of three patients were less than 1 cm^3^ to be unable to extract texture parameters. The PET image quality of the three cases was too bad to segment regions of interest (ROI). Eventually, 148 cases (54 PLNM and 94 non-PLNM) were enrolled in the study and randomly divided into a training dataset and a testing dataset according to the 7:3 ratio.

### ^18^F-FDG PET/CT technique

The patients were all performed with the PET-CT (Discovery PET/CT 690; GE Healthcare, Chicago, Illinois, United States) and received an injection of 3.7 MBq/kg ^18^F-FDG intravenous. The CT parameters were 3.27 mm slice thickness, 120 kV tube voltage, and 30–210 Ma. Then, with a three-dimensional acquisition mode and a matrix size of 192 × 192, PET data were captured at a speed of 1.5 min/bed (total of seven to eight beds). Using an iterative reconstruction algorithm of order subset expectation maximization, the PET image was reconstructed with twice iteration, 24 subsets and 6.4 mm Gaussian filter. In the AW4.5 workstation (GE Healthcare), and all PET images were transferred. The conventional metabolic parameters of tumors in ^18^F-FDG PET images for all patients consisted of total pathological glycolysis (TLG), the metabolic tumor volume (MTV), SUV_max_, SUV_peak_, and SUV_mean_. SUV corrected for body weight and was measured automatically using a threshold of 42% SUV_max_ from the ROIs.

### Immunohistochemical analysis

Department of Pathology in our hospital prepared all paraffin sections for cervical cancer. IHC staining was performed by Leica BOND MAX™ (Leica Biosystems). Goat anti-human COX-2 (1:400 dilution) polyclonal primary antibodies (Abcam) were used to incubate these sections, following species-appropriate secondary antibodies and the standard procedures were performed as in the previous report (Li et al., 2021a). Tumor sections were scanned using the Pannoramic MIDI slice scanner (3DHISTECH Ltd.) forming a digital image (×400) and analyzed by QuantCenter software with the Pannoramic viewer. The whole images were scanned by the DensitoQuant software and the analysis procedure was performed as in previous reports (Yeo et al., 2015; Li et al., 2021b). The immunoreactive scoring system (IRS) was utilized to assess the expression level of COX-2 (Kim et al., 2008). The IRS was derived from the addition of staining intensity (scored on a 0–3 scale: 0, negative; 1, weakly positive; 2, moderately positive; and 3, strongly positive) and staining extent (scored on a 0–4 scale: 0, no staining; 1, 1%–25% positive 2, 26%–50% positive; 3, 51%–75% positive; and 4, 76%–100% positive tumor cells). The level of COX-2 expression was classified as a dichotomous variable for high (IRS, 4–7) or low (IRS, 0–3) expression.

### Extracting texture features of PET and immunohistochemical images

All PET images were loaded to 3D slicer (https://www.slicer.org) software 4.10.2 version. Two nuclear medicine physicians manually segmented independently the largest slice of all tumors in PET images to form 2D ROI, blinded to patient clinical information. Then, the texture features of ROI in PET images were extracted by the pyradiomics package (van Griethuysen JJMFedorov et al., 2017). The resampled voxel size was set to 1 mm × 1 mm × 1 mm to be isotropic of the image. The discretization of the grayscale was set to 25 bin width. The PET original images were transformed into eight images by the first level wavelet transform. Then the texture features were extracted from ROI based on PET original images and wavelet transformed images.

A pathologist randomly captured the cancer tissue area of the cervix on the digital IHC image (×20). The captured images that were native red/green/blue (RGB) images were converted to greyscale before computing the texture features (Kather et al., 2016). Then the same texture features as PET images were extracted with 3D slicer.

### Dimensionality reduction of texture features

All texture feature parameters were standardized using the Z-score method. In the training dataset, with 10-fold cross-validation, the least absolute shrinkage and selection operator (LASSO) algorithm was used to filter clinical features, the conventional metabolic parameters, and texture features derived from PET images that could be used to predict PLNM and COX-2 expression. A classical metabolic parameter was generated using a linear combination of selected texture features of PET images of non-zero coefficient after dimensionality reduction. Afterward, both of them were weighted by their respective coefficients to establish the radiomics score (Rad-score) (Huang et al., 2016). The Rad-score was utilized to construct machine learning models to prognosticate PLNM and COX-2 expression.

### Establishing and testing machine learning model

The Rad-score (PLNM) in the training dataset was utilized to establish the PLNM model for predicting PLNM with logistic regression algorithm. And the Rad-score (COX-2) was the parameter for establishing the COX-2 model. The PLNM and COX-2 models in the testing dataset were tested independently.

### Statistical analysis

The Mann-Whitney U test (continuous variables) or the Pearson chi-square test (rank variables) was used to evaluate the distribution of the clinical feature between the training and testing dataset. The correlation of selected PET texture features and the corresponding IHC texture images was analyzed with the Spearman correlation method. The differences in Rad-score (PLNM) between the PLNM group and non-PLNM group in all datasets and Rad-score (COX-2) between the COX-2 high expression group and COX-2 low expression group were analyzed with the Wilcoxon test. To evaluate the results of PLNM and COX-2 models, the ROC curve was used. All data processing, establishing machine learning models, and statistical analysis were performed with R software version 3.5.1 or SPSS software version 25.0 (IBM Corp., Armonk, NY, United States). A two-tailed *p* < 0.05 was considered statistically significant in all statistical analysis.

## Results

### The distribution of clinical characteristics of patients

The basic clinical characteristics of patients were summarized in Table 1. With a median age of 51 years, 104 patients were randomly assigned to the training dataset, containing 38 patients with PLNM. COX-2 was highly expressed in 60 patients in the training dataset. There were 44 patients with a median age of 53 years old in the testing dataset. Sixteen of them were PLNM positive. In the testing dataset, there were 24 patients with COX-2 high expression. Statistical analysis showed that there was no statistically significant difference in the distribution of all clinical features between the training and the testing dataset (*p* > 0.05).

**TABLE 1:** Patient characteristics.

	Training dataset (*N* = 104)	Testing dataset (*N* = 44)	*p* value
Age	51 (33–64)	53 (35–74)	0.266
Stage			
IA	27	11	0.743
IB	43	21	
IIA	34	12	
Differentiation			0.482
Well	24	12	
Moderate	65	23	
Poor	15	9	
PLNM			1.000
No	66	28	
Yes	38	16	
COX-2			0.856
Low expression	44	20	
High expression	60	24	
WBC (×10^9^/L)	6.106	5.784	0.272
NEU (%)	60.622	59.048	0.537

### Filtering and integration of features

**FIGURE 2:**
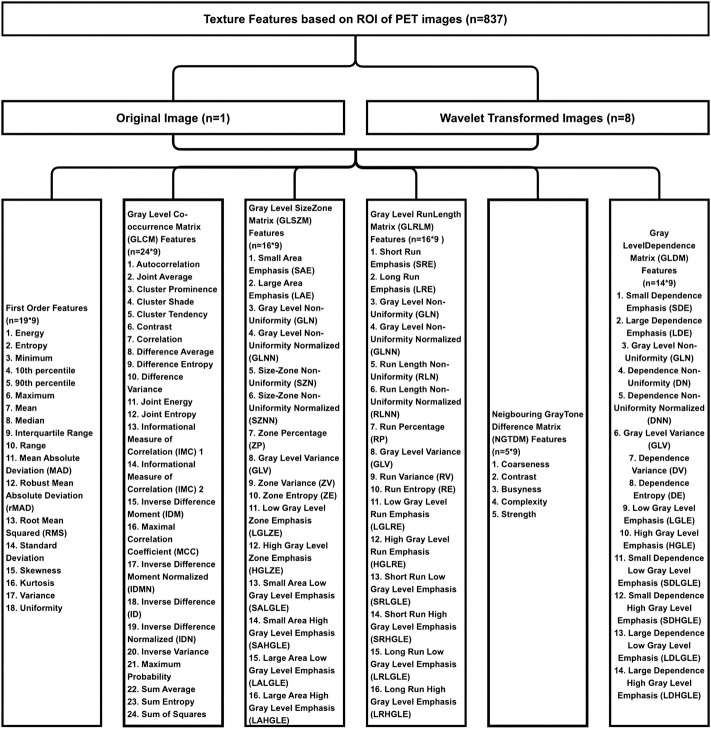
The result of extracting texture feature parameters of PET images. Eight hundred and thirty-seven texture features were extracted from the ROI in the PET image, including the first order features (*n* = 18), gray level co-occurrence matrix (GLCM) features (*n* = 24), gray level size zone matrix (GLSZM) features (*n* = 16), gray level run length matrix (GLRLM) features (*n* = 16), neigbouring gray tone difference matrix (NGTDM) features (*n* = 5), gray level dependence matrix (GLDM) features (*n* = 14), and wavelet features derived from one level of wavelet decomposistions yielding eight derived images (*n* = 93*8).

A total of 837 texture features were extracted from the ROI in the PET image, including the First Order Features (*n* = 18), Gray Level Co-occurrence Matrix (GLCM) Features (*n* = 24), Gray Level Size Zone Matrix (GLSZM) Features (*n* = 16), Gray Level Run Length Matrix (GLRLM) Features (*n* = 16), Neigbouring Gray Tone Difference Matrix (NGTDM) Features (*n* = 5), Gray Level Dependence Matrix (GLDM) Features (*n* = 14) and Wavelet Features derived from one level of Wavelet decomposistions yielding eight derived images (*n* = 93*8). The detailed texture feature parameters of PET images were shown in Figure 2. The same 837 texture features were extracted based on the IHC images.

**FIGURE 3:**
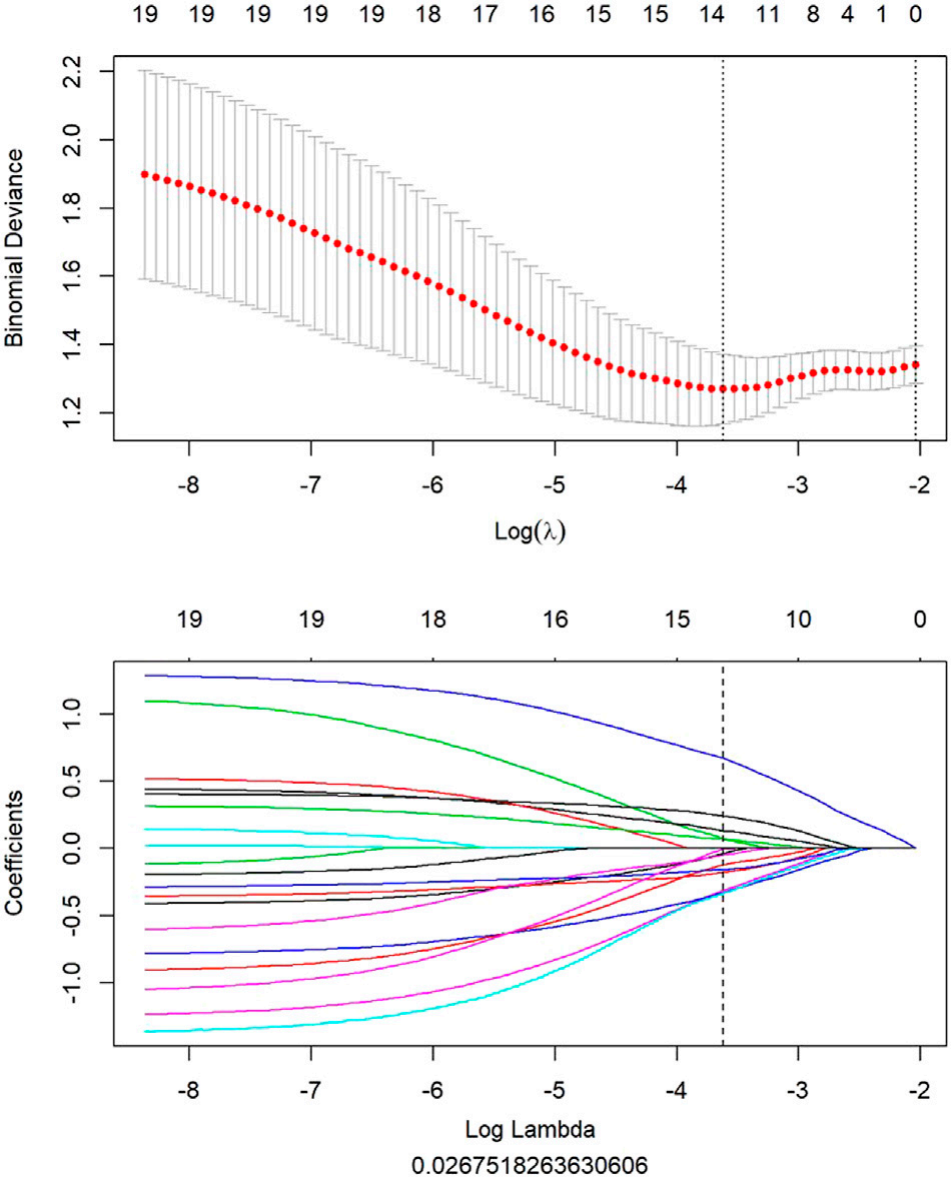
All features of PET image selected to predict PLNM by LASSO algorithm. When lambda was 0.027 in predicting PLNM with the LASSO algorithm, 14 most informative features were reserved to predict PLNM in the training dataset.

**FIGURE 4:**
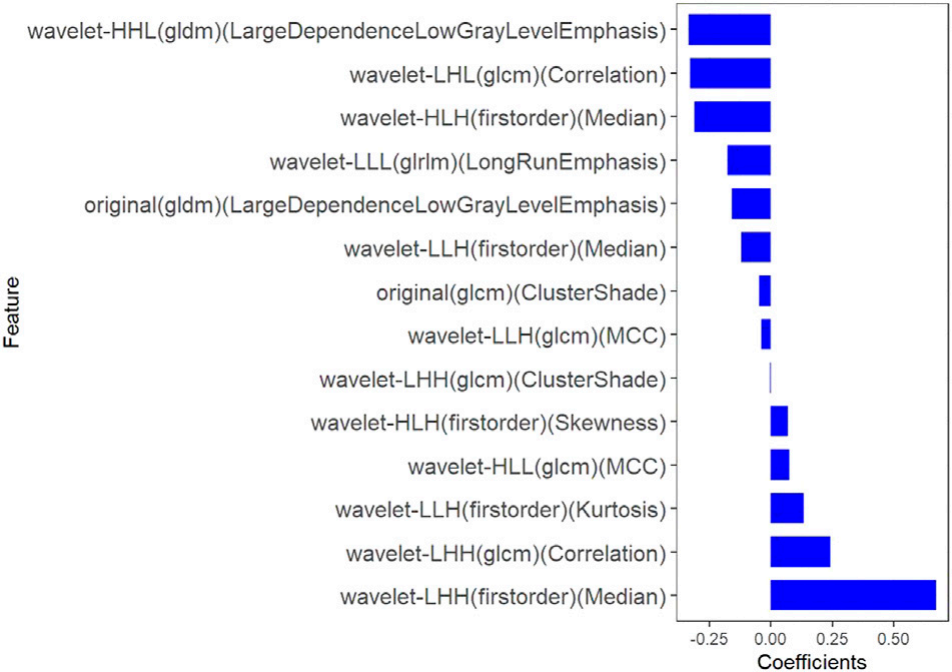
The coefficients histogram of texture features was selected to predict PLNM. Using the LASSO model, 14 corresponding texture features were selected to predict PLNM.

All clinical features, conventional metabolic parameters, and texture features derived from PET images were selected to predict PLNM. When Lambda was 0.027 in predicting PLNM with the LASSO algorithm, the 14 most informative features were reserved in the training dataset (Figure 3). And minimal binomial deviation for predicting PLNM was acquired with the 14 reserved features. Figure 4 showed that the coefficients of the reserved texture feature were used to predict PLNM with logistic regression algorithm. Partial regression coefficients were negative for nine of the reserved features and positive for five of the features. Then the reserved features were multiplied by their partial regression coefficients and linearly integrated into Rad-score (PLNM). The Rad-score (PLNM) was used to establish the PLNM model with logistic regression algorithm.

Rad-score (PLNM) = − 0.038 * wavelet-LLH-GLCM MCC
−0.177 * wavelet-LLL-GLRLM LongRunEmphasis
+0.076 * wavelet-HLL-GLCM MCC
−0.334 * wavelet-HHL-GLDM LargeDependenceLowGrayLevelEmphasis
+0.671 * wavelet-LHH-Firstorder Median
−0.311 * wavelet-HLH-Firstorder Median
+0.133 * wavelet-LLH-Firstorder Kurtosis
−0.327 * wavelet-LHL-GLCM Correlation
−0.048 * original-GLCM ClusterShade
+0.242 * wavelet-LHH-GLCM Correlation
−0.121 * wavelet-LLH-Firstorder Median
+0.069 * wavelet-HLH-Firstorder Skewness
−0.158 * original-GLDM LargeDependenceLowGrayLevelEmphasis
−0.004 * wavelet-LHH-GLCM ClusterShade − 0.641.

**FIGURE 5:**
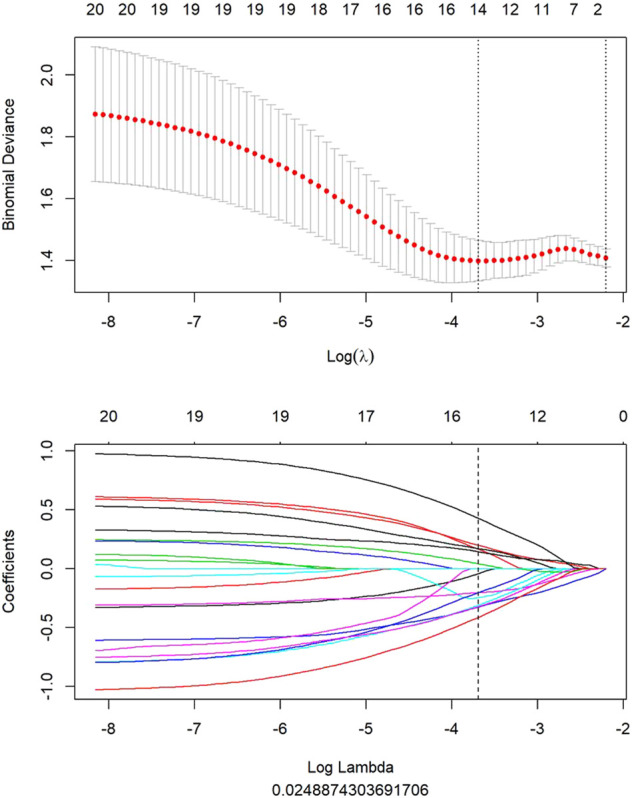
All features of PET image selected to predict COX-2 by LASSO algorithm. When lambda was 0.025 in predicting PLNM with the LASSO algorithm, 14 most informative features were reserved to predict PLNM in the training dataset.

**FIGURE 6:**
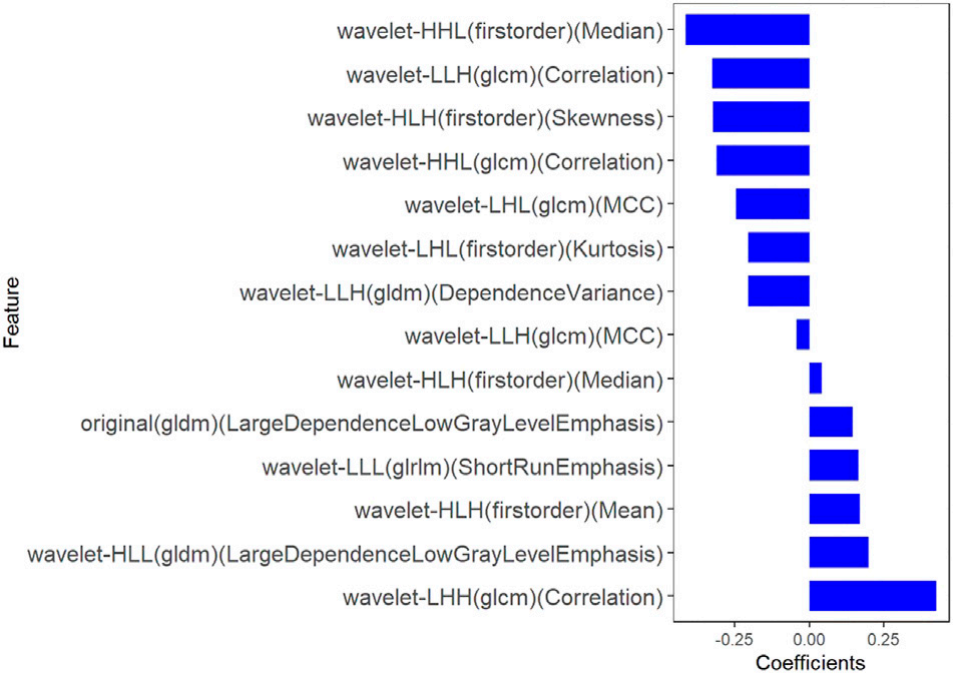
The coefficients histogram of texture features was selected to predict COX-2 expression. Using the LASSO model, 14 corresponding texture features were selected to predict COX-2 expression.

Fourteen features were selected to predict COX-2 by LASSO 10-fold cross-validation in the training dataset (Figure 5). The histogram showed the coefficients of the reserved features in Figure 6. Partial regression coefficients were negative for eight of the reserved features and positive for six of the features. Rad-score (COX-2) was integrated according to the calculation formula below. The Rad-score (COX-2) was used to establish the COX-2 model with logistic regression algorithm.

Rad-score (COX-2) = − 0.044 * wavelet-LLH-GLCM MCC
−0.326 * wavelet-LLH-GLCM Correlation
−0.245 * wavelet-LHL-GLCM MCC
+0.165 * wavelet-LLL-GLRLM ShortRunEmphasis
+0.144 * original-GLDM LargeDependenceLowGrayLevelEmphasis
+0.198 * wavelet-HLL-GLDM LargeDependenceLowGrayLevelEmphasis
−0.206 * wavelet-LHL-Firstorder Kurtosis
+0.17 * wavelet-HLH-Firstorder Mean
+0.042 * wavelet-HLH-Firstorder Median
−0.31 * wavelet-HHL-GLCM Correlation
−0.205 * wavelet-LLH-GLDM DependenceVariance
−0.324 * wavelet-HLH-Firstorder Skewness
+0.427 * wavelet-LHH-GLCM Correlation
−0.415 * wavelet-HHL-Firstorder Median + 0.324.

### Distribution differences of rad-scores in all datasets

**FIGURE 7:**
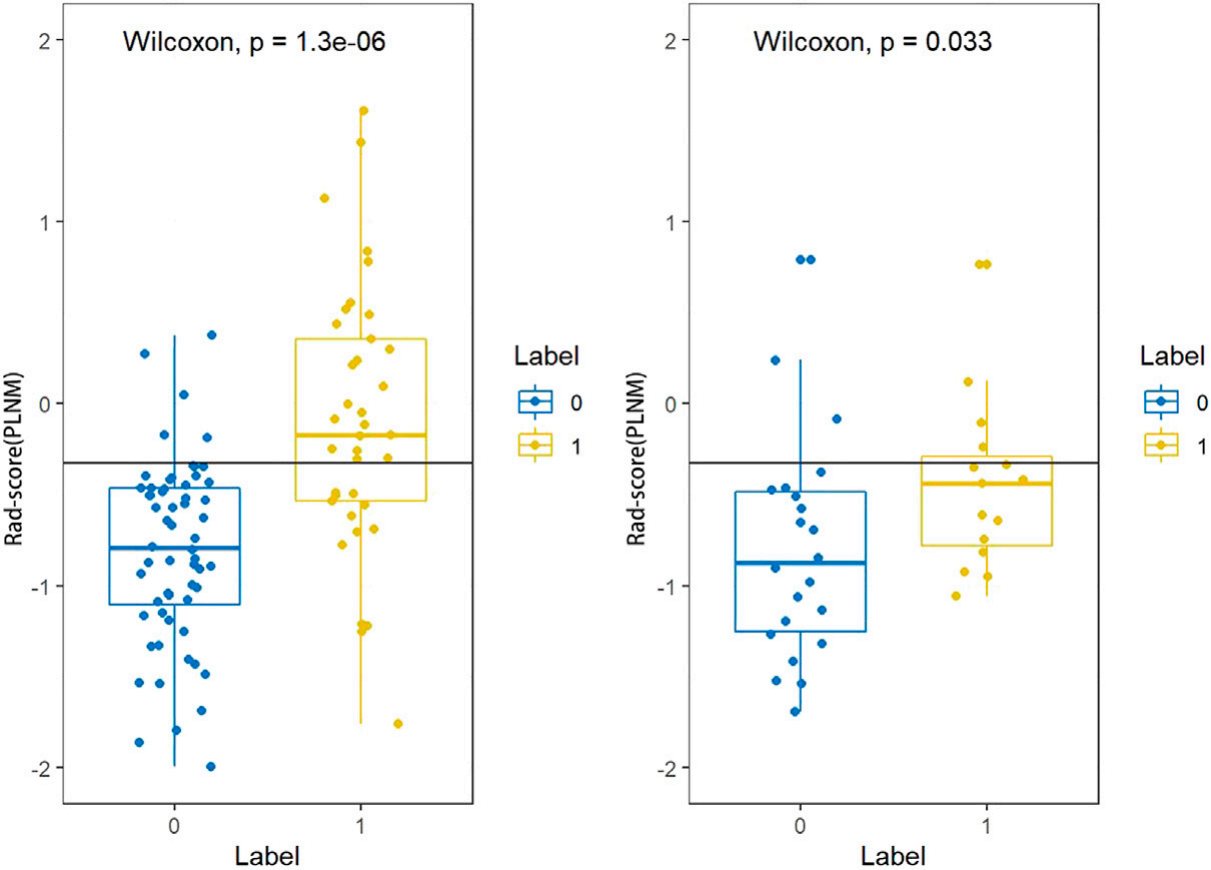
Rad-score (PLNM) distribution difference between PLNM negative subgroup and positive subgroup in the training dataset and testing dataset. The rad-score (PLNM) of patients with PLNM was higher than the rad-score (PLNM) of patients with non-PLNM in the training dataset and testing dataset (*p* < 0.001, *p* < 0.05, respectively).

**FIGURE 8:**
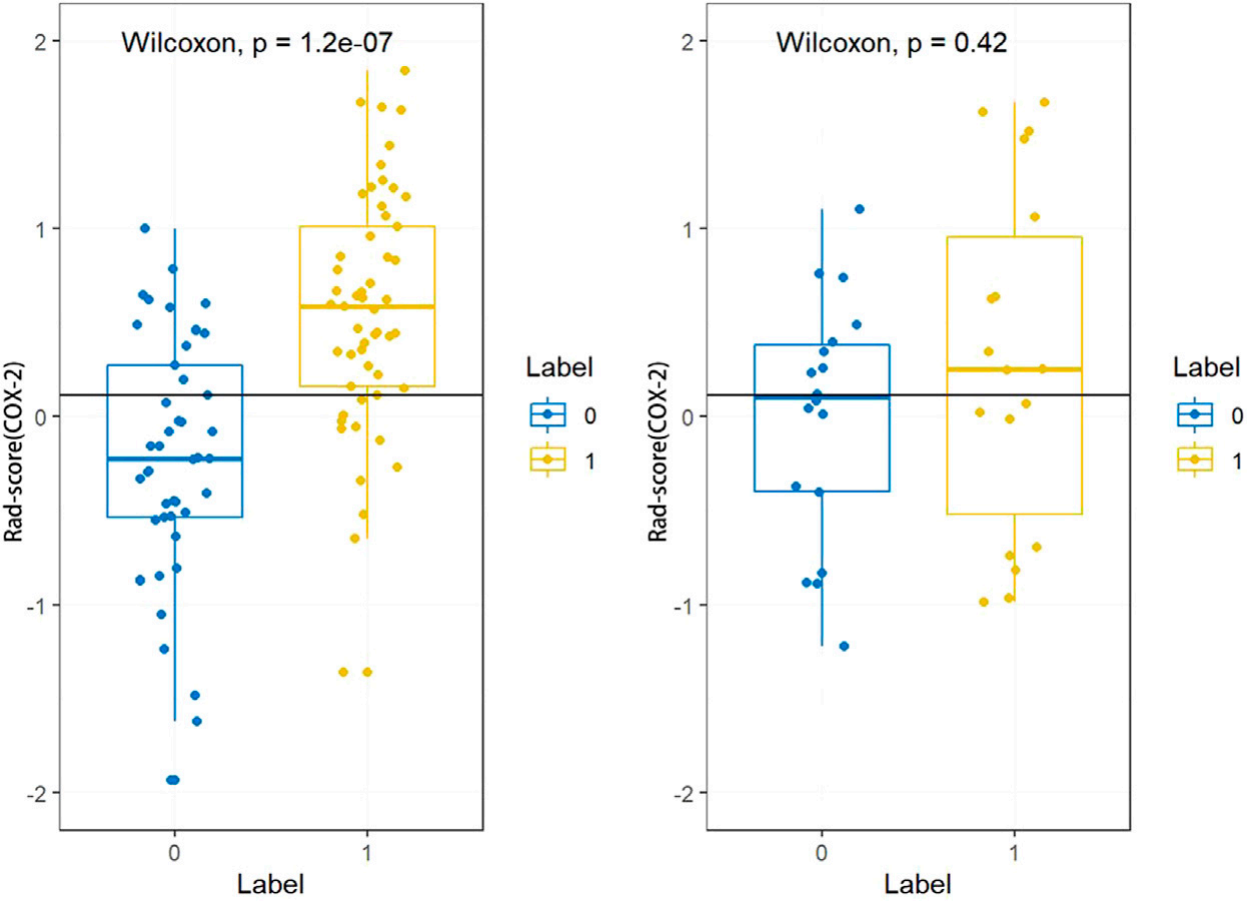
Rad-score (COX-2) distribution difference between COX-2 low expression subgroup and high expression subgroup in the training dataset and testing dataset. Rad-score (COX-2) of patients with high expression of COX-2 was higher than that with low expression of COX-2 in the training dataset (*p* < 0.001). In the testing dataset, there was no significant difference in Rad-score (COX-2) between patients with high COX-2 expression and patients with low COX-2 expression (*p* < 0.05).

The Rad-score (PLNM) of patients with PLNM was higher than the Rad-score (PLNM) of patients with Non-PLNM in the training dataset and testing dataset (*p* < 0.001, *p* < 0.05, respectively) (Figure 7). And the Rad-score (COX-2) of patients with high expression of COX-2 was higher than that with low expression of COX-2 in the training dataset (*p* < 0.001). Whereas, in the testing dataset, there was no statistically significant difference in Rad-score (COX-2) between patients with high COX-2 expression and patients with low COX-2 expression (*p* < 0.05) (Figure 8).

### Evaluation of machine learning models

**FIGURE 9:**
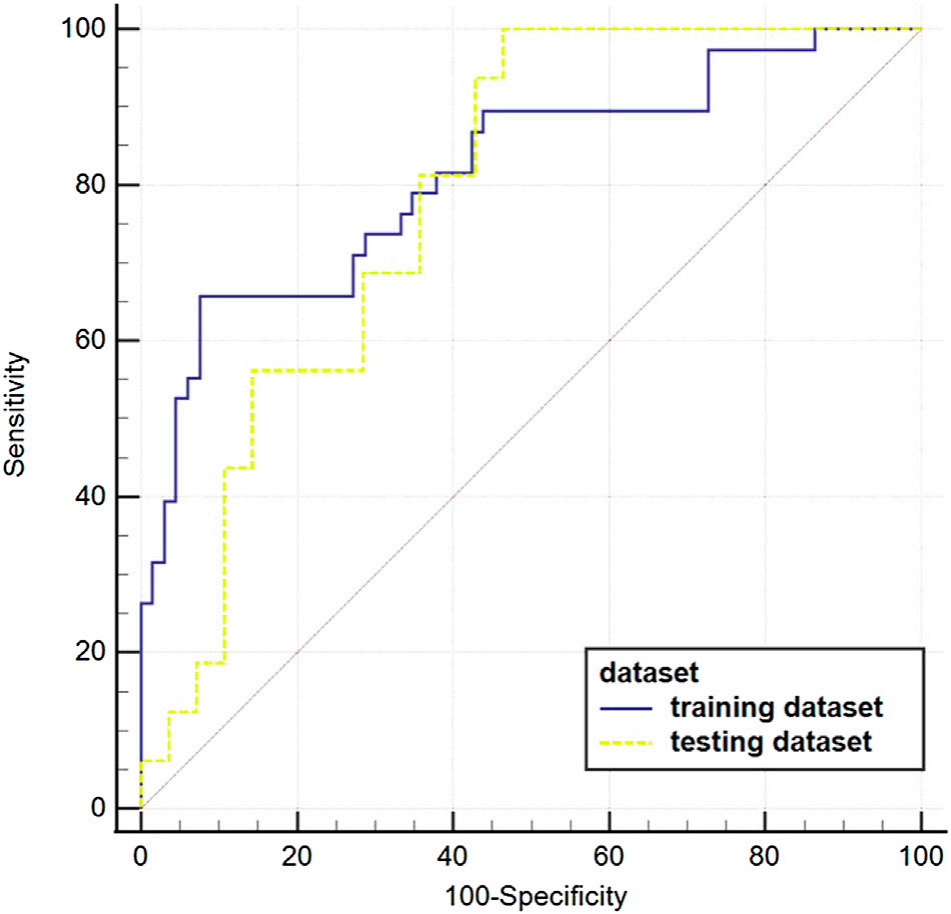
The ROC curves of the PLNM model in the training dataset and testing dataset. The blue ROC curve represents the training dataset; the yellow ROC curve represents the testing set. The AUC value of each model that was aimed to predict PLNM was 0.817 (*p* < 0.001) in the training dataset, and 0.786(*p* < 0.001) in the testing dataset.

**FIGURE 10:**
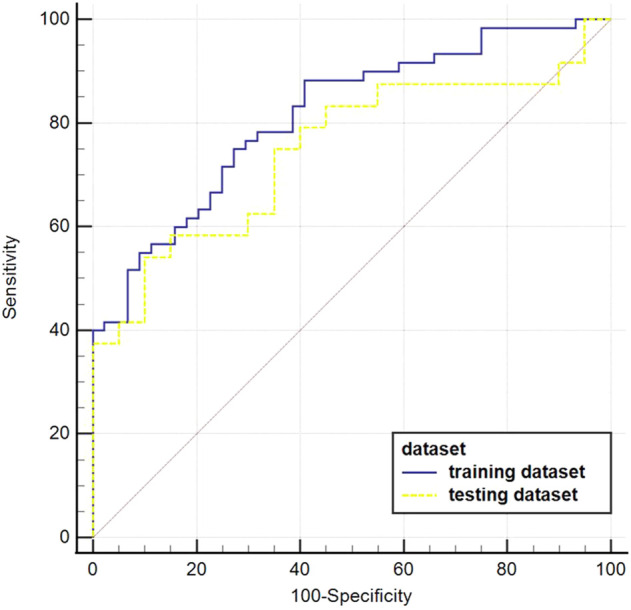
The ROC curves of the COX-2 model in the testing dataset. The blue ROC curve represents the training dataset, the yellow ROC curve represents the testing set. Both of them behaved well in predicting COX-2 expression levels in the training dataset and testing dataset (AUC = 0.814, *p* < 0.001; AUC = 0.748, *p* = 0.001, respectively).

The AUC value of the machine learning model was shown in Figure 9 which was aimed to predict PLNM was 0.817 (*p* < 0.001) in the training dataset, and 0.786 (*p* < 0.001) in the testing dataset. And the COX-2 model also behaved well for predicting COX-2 expression levels in the training and testing dataset (AUC = 0.814, *p* < 0.001; AUC = 0.748, *p* = 0.001) (Figure 10). The sensitivity (Sen) value of the PLNM model was 65.8% in the training dataset, and 100.0% in the testing dataset. The specificity (Spe) value of the COX-2 model was 72.7% in the training dataset, and 90.0% in the testing dataset (Table 2).

**TABLE 2:** Evaluation of machine learning model prediction in the training and testing dataset.

Model	Training dataset				Testing dataset			
	AUC (95% CI)	*p*	Sen	Spe	AUC (95% CI)	*p*	Sen	Spe
PLNM model	0.817 (0.730–0.886)	<0.001	0.658	0.924	0.786 (0.636–0.895)	<0.001	1.000	0.538
COX-2 model	0.814 (0.726–0.884)	<0.001	0.750	0.727	0.748 (0.594–0.866)	0.001	0.542	0.900

AUC, area under the curve; Sen, sensitivity; Spe, specificity; CI, confidence interval.

### The correlation of texture features derived from PET image with same texture features of immunohistochemical image

Table 3 showed the correlation between the texture features derived from ROI in the PET image selected to predict PLNM and the same texture features of the IHC image by Spearman correlation analysis. Only the wavelet-LHH (glcm) (ClusterShade) derived from the PET image was slightly correlated with the same feature of the IHC image (r = −0.165, *p* < 0.05). The correlation of texture features of the PET image selected to predict the COX-2 expression level with the IHC image’s same texture features as illustrated in Table 4. There was a weak correlation that wavelet-LLL (glrlm) (LongRunEmphasis) derived from the ROI of the PET image correlated with the same feature of the IHC image (r = 0.238, *p* < 0.05).

**TABLE 3:** Correlation of PET texture parameters selected to predict the PLNM with same texture parameters of IHC image.

	Feature		Spearman test	
Filter	Class	Name	r	*p*
Wavelet-LLH	GLCM	MCC	−0.157	0.057
Wavelet-LLL	GLRLM	Long Run Emphasis	−0.002	0.980
Wavelet-HLL	GLCM	MCC	0.024	0.772
Wavelet-LHH	First order	Median	−0.025	0.767
Wavelet-HLH	First order	Median	−0.006	0.946
Wavelet-LLH	First order	Kurtosis	−0.070	0.400
Wavelet-LHL	GLCM	Correlation	0.026	0.754
Wavelet-HHL	GLDM	LDLGLE	0.085	0.302
Original	GLCM	Cluster Shade	0.115	0.163
Wavelet-LHH	GLCM	Correlation	−0.016	0.849
Wavelet-LLH	First order	Median	−0.008	0.920
Wavelet-HLH	First order	Skewness	−0.144	0.080
Original	GLDM	LDLGLE	−0.155	0.060
Wavelet-LHH	GLCM	Cluster Shade	−0.165*	0.045

GLCM, gray level co-occurrence matrix; MCC, maximum correlation coefficient; GLRLM, gray level run length matrix; GLDM, gray level dependence matrix; LDLGLE, large dependence low gray level emphasis. * indicating significant correlation.

**TABLE 4:** Correlation of PET texture parameters selected to predict the COX-2 expression level with same texture parameters of IHC image.

	Feature		Spearman test	
Filter	Class	Name	r	*p*
Wavelet-LLH	GLCM	MCC	−0.157	0.057
Wavelet-LLL	GLRLM	Short Run Emphasis	−0.042	0.614
Wavelet-LLH	GLCM	Correlation	0.238*	**0.004**
Wavelet-LHL	GLCM	MCC	−0.098	0.238
Wavelet-LHL	First order	Kurtosis	−0.043	0.606
Wavelet-HLH	First order	Mean	0.123	0.135
Wavelet-HLL	GLDM	LDLGLE	0.079	0.338
Wavelet-HLH	First order	Median	−0.005	0.947
Wavelet-HHL	GLCM	Correlation	0.109	0.187
Original	GLDM	LDLGLE	0.050	0.545
Wavelet-LLH	GLDM	Dependence Variance	0.047	0.568
Wavelet-HLH	First order	Skewness	−0.144	0.080
Wavelet-LHH	GLCM	Correlation	−0.016	0.849
Wavelet-HHL	First order	Median	0.114	0.168

GLCM, gray level co-occurrence matrix; MCC, maximum correlation coefficient; GLRLM, gray level run length matrix; GLDM, gray level dependence matrix; LDLGLE, large dependence low gray level emphasis. * indicating significant correlation.

The bold values represents the wavelet-LLL (glrlm) (LongRunEmphasis) derived from the ROI of PET images correlated better with the same feature of IHC images relative to other texture features (r = 0.238, *p*<0.05).

## Discussion

We provided machine learning models to study the diagnostic **value** of the textural features in PET images for predicting PLNM and performed well with good accuracy, sensitivity and specificity. Based on PET texture analysis predicting PLNM and COX-2 expression levels, this study revealed that machine learning models could assist clinical treatment of PLN in patients with early-stage cervical cancer. The rate of PLNM among patients with cervical squamous cell carcinoma stages IA-IIA was 36.54% in the training dataset and 36.36% in the testing dataset. The rate of COX-2 high expression among the patients was 57.69% in the training dataset and 54.55% in the testing dataset. The high expression of COX-2 were characteristics to predict PLNM associated with PET texture analysis and enriched level of COX-2 in the IHC images located in tumor, respectively. The Chi-square test or M-U analysis confirmed that the distribution of all clinical features was balanced between the training and testing dataset avoiding the inaccuracy and overfitting of the imbalanced feature distribution for building machine learning models.

**FIGURE 11:**
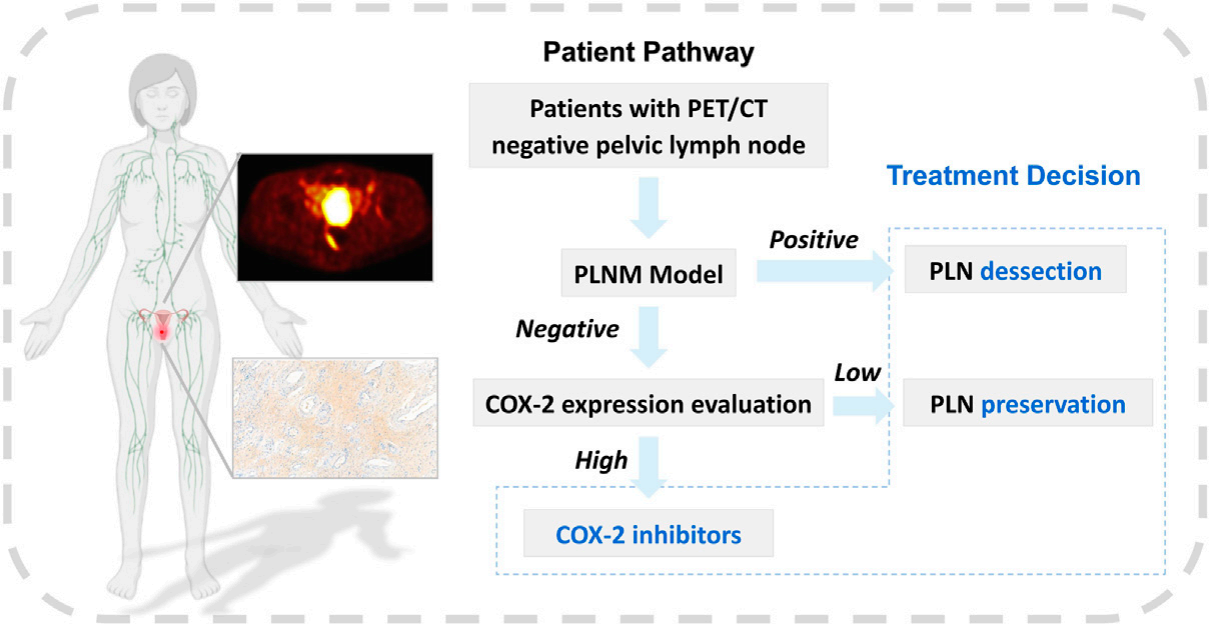
The workflow of machine learning models. The workflow of machine learning models in assisting clinical management of early-stage cervical cancer with negative PLN on PET/CT image.

The correlation between COX-2 high expression of primary tumor lesions and PLNM has been widely reported in cervical cancer (Ryu et al., 2000; Kim et al., 2003; Liu et al., 2011). The COX-2 model in this study performed well in the training and testing dataset (AUC = 0.814/0.748, *p* < 0.001/*p* = 0.001, respectively). And the specificity was 0.727 in the training dataset, and 0.900 in the testing dataset. The correlation feature of GLCM based on original and wavelet transformed images was also selected to calculate the Rad-score (COX-2). This indirectly confirmed that PET texture analysis of the primary tumor can predict that PLNM may be partly due to the high expression of COX-2. At present, the clinical evaluation of COX-2 mainly relies on IHC analysis, and the study ulitized PET/CT texture features for predicting COX-2 expression level before treatment is evaluated. We made a workflow based on the prediction of PLNM and COX-2 expression to assist the clinical management of PLN in early-stage cervical cancer (Figure 11). Kim et al. (2008) have confirmed that images with para-aortic lymph node recurrence possessed valuable expression of COX-2 attributes in cervical cancer across different patients. The prediction of the PLNM model was positive, the PLN dissection may be necessary whereas the prediction of the PLNM model was negative and the predicting COX-2 model was positive, COX-2 inhibitors were helpful for patients to control micrometastasis or recurrence of lymph nodes. Moreover, our results demonstrated the two of selected PET texture to predict PLNM and COX-2 expression that were slightly correlated with corresponding texture features from IHC images.

There are several limitations in the current study. Firstly, it was retrospective and performed at a single institution based on a small sample size. Prospective multicenter studies on automatic image acquisition and reconstruction are required to improve the process. Secondly, although we chose 2D ROI to be consistent with the 2D IHC image feature extraction method, the cross-sections of the IHC images in this study may not correspond exactly to the cross-sections of PET/CT. Further clinical studies on large-scale data sets based on the 3D printing technology are needed to achieve more accurate matching of PET images and pathological images to fully address this question. Thirdly, further test-retest studies and more standardized workflow are needed to assess feature robustness of PLNM for better generalization.

## Conclusion

In conclusion, combining PET/CT texture analysis to predict PLNM and COX-2 expression can improve the predictive ability of machine learning models for PLNM trends in PLN-negative patients. In addition, the correlation between the texture features of PET images and the corresponding texture features of IHC images provides a reasonable explanation that the texture features of the primary tumor on PET images can predict PLNM. Based on this machine learning model integrating PET/CT radiomic and IHC pathomic features, it is expected to provide guidance for the treatment strategy of negative pelvic lymph node cervical cancer in the near future.

## Data Availability

The raw data supporting the conclusion of this article will be made available by the authors, without undue reservation.

## Abbreviations

AUC, area under the curve; COX-2, cyclooxygenase-2; CT, computed tomography; FIGO, federation international of gynecology and obstetrics; GLCM, grey-level co-occurrence matrix; GLDM, gray level dependence matrix; GLRLM, gray level run length matrix; GLSZM, gray level size zone matrix; IHC, immunohistochemical; IRS, immunoreactive scoring system; LASSO, least absolute shrinkage and selection operator; LNM, lymph node metastasis; MTV, metabolic tumor volume; NGTDM, neigbouring gray tone difference matrix; PET, positron emission tomography; PLN, pelvic lymph node; PLNM, pelvic lymph node metastasis; Rad-score, radiomics score; ROC, receiver operating characteristic; ROI, region of interest; SUV, standardized uptake value; TLG, total pathological glycolysis; 18F-FDG, 18F-fluorodeoxyglucose.
